# Identification of Patients at Elevated Cancer Risk through a Community-Based Genetic Testing Program

**DOI:** 10.1245/s10434-025-17820-w

**Published:** 2025-08-06

**Authors:** Danielle Brabender, Emily Siegel, Julie O. Culver, Jacob G. Comeaux, Anjali Date, Amanda Woodworth

**Affiliations:** 1 Department of Surgery, Keck School of Medicine of USC, University of Southern California, Los Angeles, CA USA; 2 Department of Medicine, Keck School of Medicine of USC, University of Southern California, Los Angeles, CA USA; 3 Department of Radiology, Henry Mayo Newhall Hospital, Santa Clarita, CA USA; 4 Department of Surgery, Henry Mayo Newhall Hospital, Santa Clarita, CA USA

**Keywords:** Hereditary cancer, Genetic testing, Community-based screening program, Cancer detection, Cancer risk reduction

## Abstract

**Background:**

Only a subset of individuals meeting National Comprehensive Cancer Network (NCCN) guidelines for genetic screening of hereditary cancers are being offered testing. A community-based screening program has the potential to expand access, identify critical screening opportunities among those testing positive, and have potential downstream clinical effects.

**Methods:**

We conducted a retrospective review of women who underwent mammography and genetic testing at a community hospital between August 2020 and May 2023. For those testing positive for a pathogenic/likely pathogenic (P/LP) variant, potential cancer screening and surgical recommendations were identified utilizing NCCN guidelines.

**Results:**

A total of 14,192 women were screened, with 3224 (23%) meeting NCCN criteria. Of these patients, 50.3% opted for testing and 7.6% were found to have P/LP variants, encompassing 18 different genes. The genes with the highest prevalence of P/PV variants included CHEK2 (26%), MUTYH (21%), BRCA2 (8%), APC I1370K (8%), and Lynch Syndrome-associated (7%). Among those positive for a P/LP variant, individuals were identified to be at an increased risk for at least one form of cancer, including breast (52%), colon (45%), ovarian (31%), pancreas (28%), melanoma (20%), endometrial (11%), urologic (7%), and stomach/small bowel (7%), leading to a potential change in screening and risk-reducing surgery recommendations. For the total population tested, 5.3% of women qualified for MRI or earlier mammograms, and 3.4% needed earlier or more frequent screening colonoscopies.

**Conclusions:**

These findings suggest that a community-based genetic program can identify individuals at increased cancer risk who might otherwise remain undetected. These results provide opportunities to reduce morbidity/mortality through increased screening and risk-reducing procedures.

Approximately 5–10% of all cancers are associated with germline pathogenic variants;^1,2^ however, only a subset of individuals who meet the National Comprehensive Cancer Network (NCCN) guidelines for genetic screening of hereditary cancers are being offered testing.^3–5^ For example, a recent study revealed that fewer than one in five women with a history of breast or ovarian cancer, despite meeting NCCN criteria, had undergone genetic testing.^6^ Potential factors contributing to this care gap include provider awareness, lack of insurance coverage, financial burden, racial bias, limited access to genetic counseling services, and geographic barriers.^3,7,8^

One solution to this disparity would be the implementation of community-based genetic screening programs, which can potentially expand access to genetic screening, minimize bias for those being offered testing, and identify critical screening and procedural opportunities for individuals who test positive.^9,10^ Currently, genetic screening is predominantly offered in hospitals or primary care settings and relies heavily on the provider’s knowledge of when screening is indicated, which leaves a significant portion of undetected individuals with a hereditary predisposition.^5,11,12^ The case-by-case approach to genetic testing can be problematic because it depends on the provider consistently identifying eligible patients, following the appropriate screening guidelines, and subsequently ordering tests or referring patients to a genetic counselor.^13–15^ This process also does not account for individual-level barriers, such as fear of genetic results, distrust of the medical system, and perceived cost*.*^16,17^ As a result, there remains a substantial gap in care, underscored by the fact that the majority of individuals diagnosed with a hereditary cancer syndrome have already been diagnosed with cancer.^6,11,18^

A community genetic screening program, in which all patients within a health system are automatically screened and offered genetic testing as part of their routine medical care, is cost effective and expands access to genetic testing.^12,13,19,20^ This approach is critical because diagnosing hereditary cancer syndromes in unaffected individuals provides an opportunity to prevent cancer-related morbidity and mortality through timely screening and consideration of risk-reducing prophylactic surgeries.^8,10,21–24^ While previous population-based genetic screening programs have often relied on biobanks and faced recruitment challenges, innovative programs that integrate genetic screening into existing healthcare practices could address these limitations.^25–28^

Thus, the objective of our study was to evaluate the outcomes of implementing a community-based genetic screening program within a women’s imaging center, with the goal of identifying hereditary cancer syndromes prior to a cancer diagnosis. We hypothesized that implementing a community-based screening program would be feasible and that actionable genetic findings would be identified. We also sought to quantify, amongst the proportion of patients with positive results, what the potential downstream effects on clinical management would be. This is particularly important given the substantial burden cancer places on the United States (US) healthcare system and the increasing need to develop innovative and holistic approaches to expand access to genetic testing.

## Methods

To establish the genetic screening program, we included all women who underwent screening mammography between 1 August 2020 and 2 May 2023 at a high-volume community women’s imaging center, Sheila R. Veloz Breast Center at Henry Mayo Newhall Hospital, in Santa Clarita, CA, located 30 miles from Los Angeles. These patients were screened through a clinical application tool offered by a commercial genetic testing vendor (AmbryCare) to identify those who met NCCN criteria for cancer genetic testing.^29^ This third-party vendor was chosen by the mammography center given its utilization of NCCN criteria for screening, comprehensive virtual pretest education, and automatic notification system. Those who had not previously undergone testing and met NCCN criteria were offered genetic testing, which was ordered by the radiologist (AD).

The genetic screening program involved pretest education through a third-party vendor, with an artificial intelligence (AI) chatbot and videos for those who opted to participate, after which they underwent a 34-gene panel test (see Appendix A). Results of the genetic testing were then sent to the ordering provider and imaging center coordinator. If the results were negative or identified one or more variants of uncertain significance, the patient was sent a letter regarding their status by the Breast Cancer Navigator and tracked to confirm if the results were opened. Patients testing positive for a P/LP variant were called with results and referred to third-party genetic counseling offered by the genetic testing vendor. Of note, if a patient had negative results but was identified to have an elevated remaining lifetime risk of developing breast cancer ≥20% according to the Tyrer–Cuzik Score (version 8), they were notified and referred to a high-risk clinic for enhanced breast cancer surveillance.

We then conducted a retrospective descriptive analysis of data collected by the genetic screening program for patients identified to have positive pathogenic mutation test results. This analysis was performed using the most recent NCCN guidelines for genetic assessment available as of June 2023 (Colorectal: version 1.2023; Breast/Ovarian/Pancreatic: version 3.2023).^30,31^ The primary objective was to determine the potential clinical impact of identifying genetic mutations by determining the probable changes in cancer screening or risk-reducing surgeries for each patient. For those with a positive result, we considered their unique mutation characteristics, such as specific alleles and whether they were heterozygous or homozygous, when determining their individual cancer risk and potential changes to their management plan, including screening and procedural recommendations (Table 1).

**Table 1:**
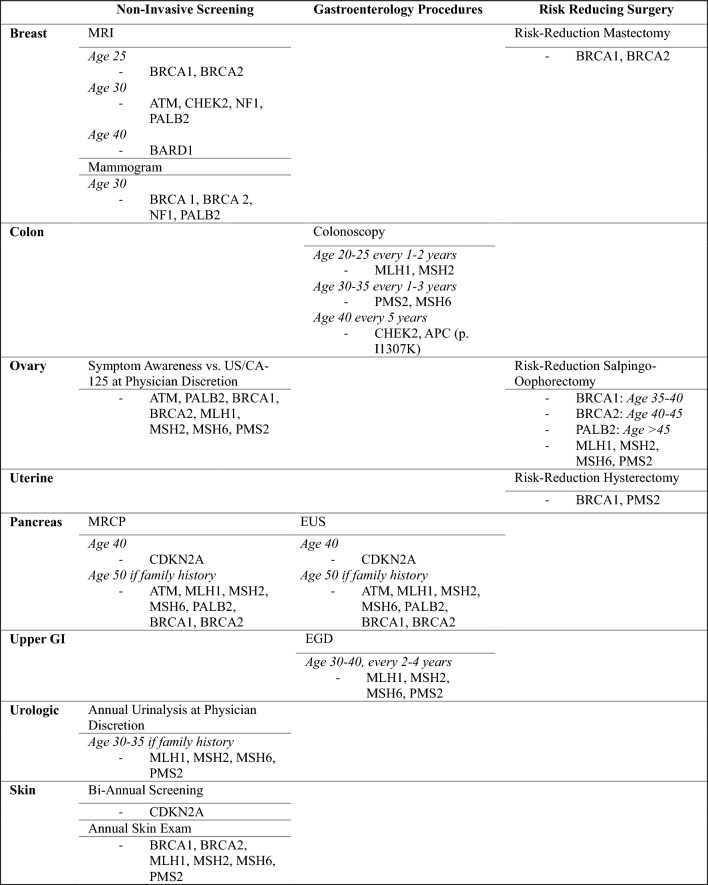
Gene-specific NCCN management considerations

*NCCN* National Comprehensive Cancer Network, *MRI* magnetic resonance imaging, *GI* gastrointestinal, *MRCP* magnetic resonance cholangiopancreatography, *EUS* endoscopic ultrasound, *EGD* esophagogastroduodenoscopy

The increased screening group in this study consisted of non-invasive imaging (e.g., magnetic resonance imaging [MRI] for breast cancer), physical examinations (e.g., skin checks for skin cancer), and symptom awareness (e.g., bloating/pelvic pressure for ovarian cancer). The procedure groups were categorized into invasive gastroenterology procedures (colonoscopy, esophagogastroduodenoscopy [EGD], and endoscopic ultrasound [EUS]) and risk-reducing surgeries (mastectomy, hysterectomy, and salpingo-oophorectomy). Descriptive statistics were expressed as frequencies and percentages for categorical variables, and all continuous nonparametric data were described as median (interquartile range). Statistical analysis was performed using SPSS v27.0 (IBM^®^ Corporation, Armonk, NY, USA).

## Results

**Fig. 1:**
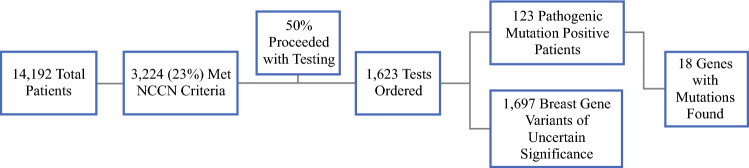
Patient selection flowchart and genetic testing outcomes. *NCCN* National Comprehensive Cancer Network

**Fig. 2:**
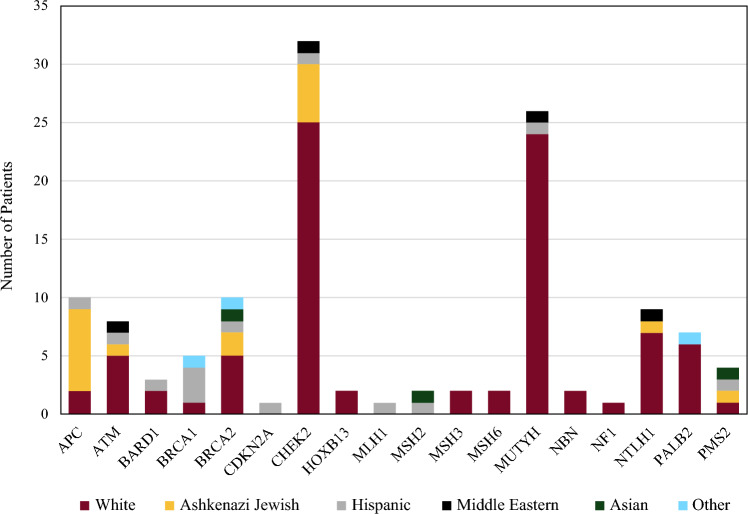
Ethnicity and prevalence of patients with pathogenic/likely pathogenic mutations

**Fig. 3:**
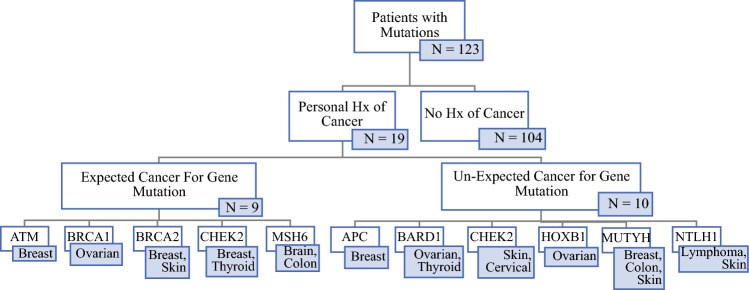
Patients with pathogenic/likely pathogenic mutations and a personal history of cancer. *Hx* history

A total of 14,192 women who underwent mammography were screened, with 23% (3224) meeting NCCN criteria. Of these patients, 50.3% (*n* = 1623) opted for testing, of whom 7.6% (*n* = 123) were found to have pathogenic/likely pathogenic (P/LP) variants in 18 different genes (Fig. 1). For those who tested positive, the median age was 55 years (28–84) and the population was largely White (non-Hispanic/non-Ashkenazi Jewish) at 69% (*n* = 87), followed by Ashkenazi Jewish at 14% (*n* = 17), Hispanic at 10% (*n* = 13), Middle Eastern at 3% (*n* = 4), Asian at 2% (*n* = 3), and other at 2% (*n* = 3) (Fig. 2). The number of patients with a pathogenic mutation and a past medical history of cancer was 19 (15%), with the most prevalent type being breast cancer (32%, *n* = 6). All patients who had a history of cancer prior to screening were over the age of 50 years, and 47% (*n* = 9) had a cancer associated with their pathogenic mutation (Fig. 3). The genes in which P/LP variants were identified with the highest prevalence were *CHEK2* at 26% (*n* = 32), monoallelic *MUTYH* at 21% (*n* = 26), *BRCA2* at 8% (*n* = 10), *APC* (I1370K) at 8% (*n* = 10), and Lynch Syndrome at 7% (*n* = 9) (Fig. 2). Of note, 58% (*n* = 19) of the *CHEK2* variants were missense mutations (p.I157T, p.S428F, and p.T476M).

**Fig. 4:**
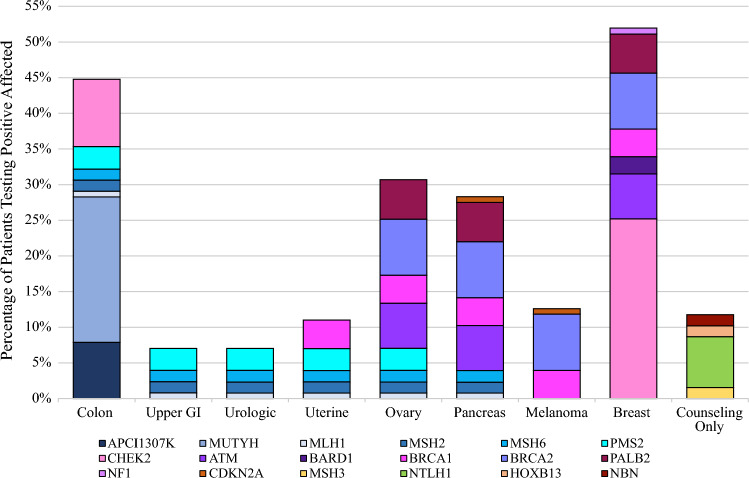
Organ systems potentially affected by gene mutations in the patient population. *GI* gastrointestinal

Among the 123 patients who tested positive for P/LP variants, an increased risk for various cancers was identified, including breast (52%), colon (45%), ovarian (31%), pancreatic (28%), melanoma (13%), endometrial (11%), urologic (7%), and stomach/small bowel cancers (7%) (Fig. 4). Since this was a female-only population, gene mutations affecting male reproductive organs (e.g., *HOXB13*) or those only influencing risk when homozygous (e.g., *NTLH1*) were categorized under Family Counseling (12%).

**Fig. 5:**
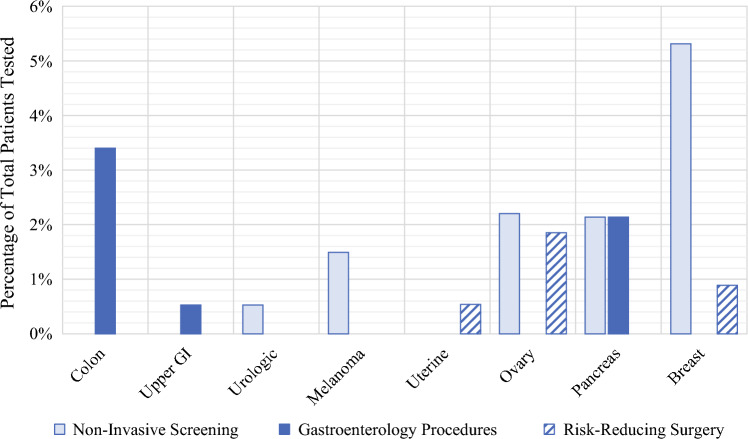
Potential change in management based on organ system. *GI* gastrointestinal

Among the entire population of 1623 patients tested, we calculated the potential impact to clinical management, including non-invasive screening, gastroenterology procedures, and risk-reducing surgeries. Within the entire population, we evaluated each organ system affected and found that 5.3% of patients had a potential increased need for breast imaging (MRI, mammogram) based on their results, and 3.4% of patients had a potential increased need for screening colonoscopies (Fig. 5). Other organs/systems affected included upper gastrointestinal (GI), with a 0.54% increased need for EGD; pancreas, with a 2.24% increased need for EUS and magnetic resonance cholangiopancreatography (MRCP); urologic system, with a 0.54% increase in non-invasive screening (e.g., urinalysis); melanoma, with a 1.5% increase in non-invasive screening; uterus, with a 0.54% increased need for risk-reducing surgery; and the ovaries, with a 2.21% increase in non-invasive screening and a 1.9% increase in risk-reducing surgery. Additionally, we evaluated the ages at which participants underwent testing, and 13 patients who were found to have a P/LP variant were eligible to initiate gene-specific cancer screening before the recommended age of population screening for the same cancer type (e.g., colonoscopy starting at age 40 years instead of 45 years). This clinical information allows patients to pursue genetic mutation-specific screening at a more appropriate time window. None of these patients had a prior history of cancer.

## Discussion

This study was unique in its evaluation of the clinical impact of a community-based genetic screening program on patient management. Among the 123 patients who tested positive, an increased risk was identified for several cancers, including breast and colon cancer. This elevated risk yields potential change to management for all patients screened, with the most notable findings for the entire population (*n* = 1623) being that there was a prospective 5.3% increase in the need for breast imaging (MRI, mammogram) and a 3.4% increase in the need for screening colonoscopies. Additionally, 13 patients were younger than the NCCN mutation-specific recommendation or the standard screening age, underscoring the potential benefit of early detection screening for these higher-risk individuals.

In our study, the use of a mammography screening unit provided a unique opportunity to universally screen all women within a local community hospital system, likely contributing to our high enrollment rate (50.3% of those meeting NCCN criteria). This contrasts with prior studies that struggled with enrollment, likely due to factors such as recruitment strategies via primary care providers, patient psychosocial barriers, distrust of the medical system, and cost.^11,13,25,32^ The level of engagement in our cohort could also potentially be due to increased patient-provider trust and engagement within a local community hospital setting compared with an outside research team, but this hypothesis would require further study prior to validation.

The ‘mainstreaming’ of genetic testing allows for critical expansion of access by meeting patients where they are, often reaching those previously missed by standard screening methods, such as patients in our study who met NCCN criteria and had a personal history of cancer but had never undergone genetic testing. The benefits of expanded access are far-reaching, providing advantages for both patients and hospital systems. For patients, the primary benefit is increased knowledge of their personal and familial risk factors, enabling them to make informed decisions about their health*.*^8,10,22,23,26^ This is exemplified by the 13 patients in our study who were identified earlier than their mutation-specific screening recommendations, giving them the option to begin essential screening, such as breast MRI and colonoscopy (compared with mammogram and Cologuard), as well as risk-reducing surgeries before a potential cancer diagnosis. The results also carry implications for family members of those with mutations as they can now be offered cascade testing.

These windows of opportunity emphasize the potential of universal genetic screening to save lives through increased screening and early intervention. A study from Australia examining the benefits of population genomic screening for young adults within a healthcare system to detect *BRCA1/BRCA2/MLH1/MSH2* gene variants found that population screening could reduce variant-attributable cancers by 28.8% and cancer deaths by 31.2% compared with targeted testing.^33^ Genetic screening also has the potential to significantly reduce healthcare costs by enabling early identification of individuals at risk for hereditary conditions, leading to timely intervention and prevention strategies*.*^33–35^ Zhang et al. reported an incremental cost-effectiveness ratio (ICER) of AUS$4038 per disability-adjusted life-year (DALY) prevented, and Teppala et al. also found germline testing for hereditary cancers were cost effective, with ICERs as low as $3012 per quality-adjusted life-year (QALY) in high-risk individuals.^33,34^ Ramdzan et al. showed that colorectal cancer genetic testing provided 1.53 additional QALYs per patient and was more cost effective than traditional screening, even dominating the immunochemical fecal occult blood test (iFOBT) in comparisons.^35^ Genetic testing also has the potential to be revenue-producing, as seen in a recent study which found that, on average, genetic testing generated $4,760,000 per year for a hospital system, with an estimated revenue of $97,000 per patient with *BRCA1/2* mutations and $71,000 per patient with Lynch syndrome.^36^

In our study, we utilized a third-party genetic testing platform due to its ease of use for patients and its alignment with NCCN screening criteria. Despite this, one of the initial concerns with using these programs is the potential risk of patients with positive results being lost to follow-up. To address this concern, our institution hired a High-Risk Breast Cancer Navigator to track patients, ensure they were notified of their results, and follow up with those who had not yet opened their results. However, using a third-party system does have limitations compared with a hospital-based genetic screening program, such as the lack of in-person pretest counseling, the lack of access to the patient’s medical records, and the ability to order targeted genetic panels. In a traditional system, pre- and/or post-test counseling is conducted by a genetic counselor, either in person, by telemedicine, or over the phone, whereas in the third-party system used in this study, a chatbot and videos guided patients through the process.

Research has shown that standard pretest genetic counseling can reduce patient worry, increase knowledge, decrease perceived risk, increase correct genetic test ordering, and prevent inappropriate services, although it has not been shown to significantly impact patient anxiety, intent to pursue testing, or improve cancer prevention behaviors.^37^ The recent MAGENTA Trial also demonstrated that omitting individualized pre- and post-test counseling for those without pathogenic variants during remote genetic testing was not inferior in terms of post-test distress.^38^ However, it is important to note that this trial was conducted primarily in a White, college-educated population undergoing a standard genetic testing panel, with dedicated staff for positive results follow-up, similar to our own population. There may be settings when a third-party testing program is adequate for a particular patient population, but further research is needed to understand the implications in a more diverse population prior to broad implementation. It is also worth noting that direct-to-consumer products for cancer genetic testing are available for patients to purchase, but providers should be aware that these products are often limited and inaccurate compared with clinical grade testing through hospital-based systems, and may not include adequate pre- and post-test education to inform patients of their genetic risks*.*^39^

These results should be taken in the context of its limitations. This was a retrospective, single-center study with a fairly homogeneous, female, well-educated, and insured patient population, which limits its generalizability to other settings. Furthermore, this patient population is likely a relatively motivated cohort, as evidenced by their compliance with screening. We did not have access to family health history data to calculate individual risk or determine why patients met NCCN inclusion criteria. Additionally, we were unable to ascertain which procedures or screenings were performed following positive results or if patients and their providers understood their results. Furthermore, since the completion of our analysis, the understanding of certain genes and their associations with cancer has evolved, which may have implications for genetic screening requirements and recommendations. For instance, *MUTYH* and many of the *CHEK2* heterozygotes are no longer considered at increased risk for developing colorectal cancer and are not recommended to undergo enhanced screening.

Some CHEK2 variants such as p.I157T have only an attenuated association with breast cancer.^40–42^ A large case-control study found only a small increase in risk (odds ratio 1.30, 95% confidence interval 1.06–1.59; *p* = 0.01), which is not considered enough to change screening recommendations based on the variant alone.^43,44^ The American College of Medical Genetics recommends that breast surveillance should be based on personalized risk assessment, including family history, to determine whether the combination of risk factors reaches the threshold for offering breast MRI screening.^44^ When offering genetic testing to women with similar ethnic background as those in our study, about half of the variants detected in CHEK2 would fall into this category; however, due to having other risk factors in combination, many of these women would have care and screening recommendations impacted by the genetic test result.

Another example is that the NCCN guidelines now recommend enhanced breast cancer screening with MRI for BARD1, RAD51C, and RAD51D carriers where they historically had not.^42^ It is important to note that the understanding of many of these genes are still in flux, and management guidelines and counseling have changed with it. Thus, it is key that management should be individualized based on multidisciplinary team recommendations that take the patient’s full medical/family history into consideration when providing counseling.

## Conclusion

These findings suggest that a community-based genetic screening program can identify individuals at increased cancer risk who might otherwise remain undetected. These results may provide opportunities to reduce morbidity and mortality through increased screening and risk-reducing procedures. Future directions to further elucidate these findings include the utilization of prospective, multicenter trials to more clearly define the effect of genetic mutation testing on overall survival, cancer diagnosis, and procedural intervention.

## Data Availability

Data used in this study are available from the corresponding author upon reasonable request.

## Appendix A

Genes included in the testing panel

1. APC
2. ATM
3. AXIN2
4. BARD1
5. BMPR1A
6. BRCA1
7. BRCA2
8. BRIP1
9. CDH1
10. CDK4
11. CDKN2A
12. CHEK2
13. DICER1
14. EPCAM
15. GREM1
16. HOXB13
17. MLH1
18. MSH2
19. MSH3
20. MSH6
21. MUTYH
22. NF1
23. NTHL1
24. PALB2
25. PMS2
26. POLD1
27. POLE
28. PTEN
29. RAD51C
30. RAD51D
31. SMAD4
32. SMARCA4
33. STK11
34. TP53
